# Supplementary material for: Patterns of Intron Gain and Loss in Fungi
Source: PLoS Biol. 2004 Nov 30;2(12):e422. doi: 10.1371/journal.pbio.0020422 (PMC532390; doi:10.1371/journal.pbio.0020422)
Supplement: Table S1 — Also available at http://genes.mit.edu/NielsenEtAl/. (4.3 MB ZIP). [file pbio.0020422.st001.zip › NielsenEtAl/html/725.html]

AN0275.1.NCU01250.1.MG07068.1.FG07129.1


```
 CLUSTAL W (1.82) Multiple Sequence Alignments - Introns Inserted


Sequence 1: NCU01250.1	298 aa
Sequence 2: FG07129.1	304 aa
Sequence 3: MG07068.1	279 aa
Sequence 4: AN0275.1	284 aa
Alignment Length: 316 aa
Number Identitical Residues: 94 aa
Alignment Score (without introns) 5237


MG07068.1 	----MDLLPWVSW~LFGWVY~FTCWSLSFYPQPILNNQRQSTTGTTIDFPLSN-CL~G1F
NCU01250.1	-MTDSTFLTSLSH~VFGWTY~FTCWSLSFYPQSILNFSRKSTSGTTIDFPLINSLA1G~F
FG07129.1 	-MTVS-FLNFLSG~LFGWVY~FFAWSASFYPQPLLNWRRRSTTGTTVDFPLIN-VL~G1F
AN0275.1  	MSQLEAFIRALSR2LLGWIY2TFCWSASFYPQPIDNYRRKATTGLAIDFPTIN-VL~G~F
          	      ::  :*  ::** *   .** *****.: *  *::*:* ::***  *    * *

MG07068.1 	FSYFIYNLALCYSPIIRSQYAARN-HGLEPTTRFNDVVFSLHGFILSCVTTSQYIPGLWT
NCU01250.1	FAYTISNIAFAYSPLIRAQYAVRY-KGLTPTVQLNDITFAFHGLILCFVTVSQYLFPRAW
FG07129.1 	AAYFSSNVAFYYSPEIRAQYAARH-KGLESTVQFNDITFALHALFLSIITTSQYLSPALW
AN0275.1  	VCYAVYTGTFLYSPVIRHQYAARHPLAPEPTVRFNDFAFALHAVVLSGLVYSQFWP-SIW
          	 .*   . :: *** ** ***.* . .  .*.::**..*::*...*. :. **:      

MG07068.1 	GFERAPVSQRPSRWMLGIITGCIVGVAAVFLFVAGREGPGT---DPARD--WCWLDV~MY
NCU01250.1	GFAPS-LGSRPSRFILGTFFGCVAGALIIIFRVWASPVRNDTSGAAVTEGGWVWLDA~IY
FG07129.1 	GFAPN-AGNRPSRFILGVAAGCITGVLFTCAIVSASPE-ND----AVYD--WVALDI~VY
AN0275.1  	GFRVS-RFQRVSKPIAGLFWGAFVAVAMVICVILVKSPDDG-----YEPLSWAWIDV0IY
          	**      .* *: : *   *.....      :      .          .*  :*  :*

MG07068.1 	AVSYVKLIVTLVKYTPQVIVNHRNRSTKGWSISQIALDFSGGLLSIAQQSIDSYLQHDWS
NCU01250.1	AVSYVKLVVTLIKYTPQVIVNYRNRSTEGWSILQILLDFGGGILSIAQQAIDSYQQGDWS
FG07129.1 	AVGYVKLIVTLIKYTPQIVANYRNQSTEGWSIFQILLDLTGGVLSVSQQAIDSYQQRDWS
AN0275.1  	TLSYVKLVITVVKYVPQAWVNHKRKSTRGWNIVQILLDFTGGVLSLAQLILDSAFQDDWS
          	::.****::*::**.**  .*::.:**.**.* ** **: **:**::*  :**  * ***

MG07068.1 	GFTGNPVKLGLANVSMIYDSIFFVQHYVLYPSSTSAKGPEAEG--------------~--
NCU01250.1	GITGNPVKFALGNVSMIYDLVFIMQHYVLYRHAAGAKADGAEGDSDLD---------~PL
FG07129.1 	GITGNPVKFSLGNISMIYDSVFIVQHYVLYRDAESTQSGERENESLLQDEERSAERS1PV
AN0275.1  	GVTGNPIKFLLSNVSIFFDLLFMVQHYILYRDAENKDKNNGPS--------------~--
          	*.****:*: *.*:*:::* :*::***:**  : . .     .                 

MG07068.1 	----LLGRDEEERRLD------
NCU01250.1	LREDVRGGGDEERRIQ------
FG07129.1 	QQDGFVRTAEKTRCIGMRLAAQ
AN0275.1  	MYSPLLSGGLPTSA--------
          	  . .
```
